# Supplementary figures and images for: Thermoelectric Properties of Mg3(Bi,Sb)2 under Finite Temperatures and Pressures: A First-Principles Study
Source: Nanomaterials (Basel). 2023 Dec 28;14(1):84. doi: 10.3390/nano14010084 (PMC10780500; doi:10.3390/nano14010084)

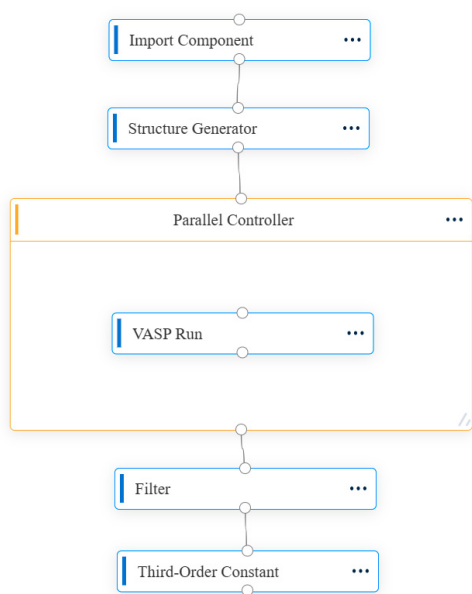

**Figure S1.** Workflow for calculating third-order constants on MatCloud.

Supplement: Supplementary file 1 [file nanomaterials-14-00084-s001.zip › nanomaterials-2747020-supplementary.pdf]
